# The role of ligand-gated conformational changes in enzyme catalysis

**DOI:** 10.1042/BST20190298

**Published:** 2019-10-28

**Authors:** Cátia Moreira, Ana Rita Calixto, John P. Richard, Shina Caroline Lynn Kamerlin

**Affiliations:** 1 Science for Life Laboratory, Department of Chemistry - BMC, Uppsala University, BMC Box 576, S-751 23 Uppsala, Sweden; 2 Department of Chemistry, University at Buffalo, SUNY, Buffalo, New York 14260-3000, U.S.A

**Keywords:** computational modeling, dianion activation, enzyme catalysis, loop dynamics, triosephosphate isomerase

## Abstract

Structural and biochemical studies on diverse enzymes have highlighted the importance of ligand-gated conformational changes in enzyme catalysis, where the intrinsic binding energy of the common phosphoryl group of their substrates is used to drive energetically unfavorable conformational changes in catalytic loops, from inactive open to catalytically competent closed conformations. However, computational studies have historically been unable to capture the activating role of these conformational changes. Here, we discuss recent experimental and computational studies, which can remarkably pinpoint the role of ligand-gated conformational changes in enzyme catalysis, even when not modeling the loop dynamics explicitly. Finally, through our joint analyses of these data, we demonstrate how the synergy between theory and experiment is crucial for furthering our understanding of enzyme catalysis.

## Introduction

Daniel Koshland proposed in 1958 that the specificity of aminoacyl *t*-RNA synthases for charging their cognate amino acids to *t*-RNA is obtained through the utilization of binding interactions between the synthase and the cognate α-amino acid side chain, to induce a change in protein structure that draws the enzyme catalytic groups into their active conformation [1]. This induced fit proposal predicted the existence of ligand-gated conformational changes years in advance of the first enzyme X-ray crystal structure determination for egg-white lysozyme reported in 1965 [2], or the observation of such a conformational change in a comparison to the X-ray crystal structures for free triosephosphate isomerase (TIM) and for TIM complexed to substrate dihydroxyacetone phosphate (DHAP) described in 1981 [3]. Ligand-gated conformational changes have been documented for many enzymatic reactions [4], but the original induced fit proposal was criticized for a lack of clarity in the rationale for utilization of substrate binding energy to drive thermodynamically unfavorable ligand-gated conformational changes [5,6]. We have examined the mechanism of action of three enzymes that undergo ligand-gated conformational changes: TIM [7,8], orotidine 5′-monophosphate decarboxylase (OMPDC) [9], and glycerol 3-phosphate dehydrogenase (GPDH) [10,11]. We describe in this review the mechanistic rationale for the utilization of the binding energy of the phosphoryl group of the substrate (or the phosphite dianion in the case of studies of substrate fragments) to drive these ligand-gated conformational changes, the common structural elements for the three enzyme conformational changes, as well as the results of ongoing computational studies to model the role of this conformational change in catalysis by TIM.

## Ligand-gated conformational changes

**Figure 1.**
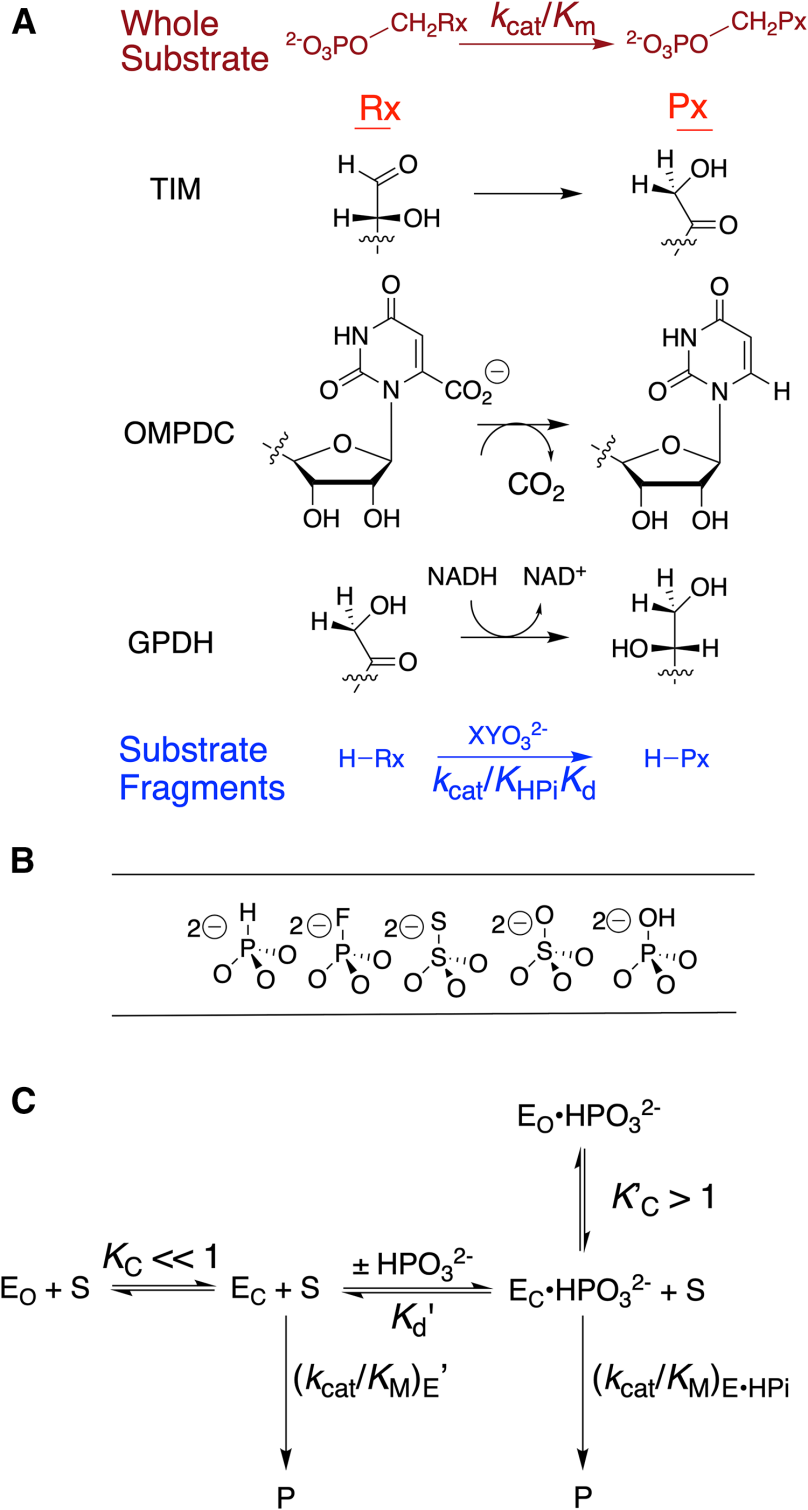
Enzyme-catalyzed reactions of whole substrates and substrate fragments. (**A**) Proton transfer, decarboxylation and hydride transfer reactions of whole substrates 
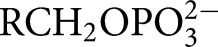
 (*k*_cat_/*K*_m_) and the substrate fragments 
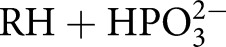
 (*k*_cat_/*K*_HPi_*K*_d_) catalyzed by TIM, OMPDC, and GPDH, respectively. ‘Rx’ and ‘Px’ denote reactant and product states, respectively. The phosphoryl group of each whole substrate provides a total 11–13 kcal·mol^−1^ stabilization of the transition states for the catalyzed reaction, while 1.0 M phosphite dianion provides a 6–8 kcal·mol^−1^ stabilization of the transition states for the catalysis of each truncated substrate fragment [13]. (**B**) Dianion activators of the reactions of the substrate fragments [10]. (**C**) The model developed to rationalize activation of TIM, OMPDC and GPDH.

TIM, OMPDC, and GPDH each undergo large ligand-gated conformational changes in catalyzing the chemically diverse set of proton transfer, decarboxylation and hydride transfer reactions shown in Figure 1A [12]. The phosphoryl group of each whole substrate for these enzymes provides a ∼12 kcal·mol^−1^ stabilization of the respective transition states [10,13]. One explanation for this stabilization is that the phosphoryl group provides a strong anchor for attachment of the substrate to the enzyme. However, large effects from the binding of a charged group are observed in the absence of the anchoring covalent attachment between the substrate and its phosphoryl group, through the phosphite dianion activation of the reaction of truncated substrate fragments, as is also shown in Figure 1A. This activation corresponds to a 6–8 kcal·mol^−1^ stabilization of the transition state for the reaction of the respective truncated substrates by the neighboring phosphite dianion fragment, and a 4–6 kcal·mol^−1^ additional stabilization from the covalent attachment of the two substrate fragments [13,14]. The striking similarity in the relative kinetic parameters for the activation of TIM, OMPDC, and GPDH by 
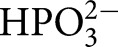
, 
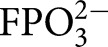
, 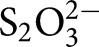, 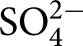, and 
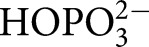
 (Figure 1B) for reactions of their respective truncated substrates reveal a similarity in the specificity of these enzymes for dianion activation [10]. These results show that the binding pocket for the whole substrates can be usefully partitioned into a catalytic site, that carries out chemistry on the bound substrate, and a dianion activation site that utilizes protein-interactions to optimize catalysis at the catalytic site [10].

The ligand-gated conformational changes undergone by TIM, OMPDC, and GPDH each conform to the Koshland's induced fit model, where the binding energy of the dianion of the phosphoryl group (or phosphite dianion in the case of the binding of the substrate fragments) is utilized to induce an enzymatic conformational change, from the inactive open protein to the catalytically active closed form. These enzymes exist mainly in the inactive open form, **E_O_**, and the binding interactions between the substrate phosphoryl group and protein loops are utilized to stabilize the active closed form, **E_C_** (Figure 1C) [12,13,15]. Enzyme activation results from the perturbation of the conformational equilibrium from 
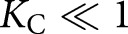
 for the unliganded form of the enzyme, to 
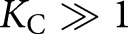
 for the liganded enzyme (Figure 1C) [12,13,15]. The energetic barrier to conversion of **E_O_** to **E_C_** represents, minimally, the energetic cost for extrusion to bulk solvent of water molecules that solvate polar active site side chains at the open enzyme, and the entropic cost for the immobilization of large flexible protein loops and smaller catalytic amino acid side chains that accompanies conversion of **E_O_** to **E_C_** [13,15,16]. The nonreacting phosphoryl group (or substrate fragments such as phosphite dianion) play the role of ‘cement’ in Figure 1C to hold the protein catalyst in a rare stiff form, with high activity for catalysis of the reaction of the bound substrate, which is trapped in a structured protein cage.

Figure 1C shows a general mechanism by which enzymes utilize the binding interactions between the protein and nonreacting substrate fragments to obtain specificity in binding their transition states with a higher affinity than substrate [5,12]. This specificity is required for powerful enzyme catalysts, because the full expression of the large transition state binding energy at the Michaelis complex would result in effectively irreversible binding of substrate and/or product [5,12]. This specificity in transition state binding may be obtained either through the utilization of the intrinsic substrate binding energy to activate the substrate for enzymatic catalysis, by the introduction of destabilizing interactions into the substrate that are relieved at the enzymatic transition state, or, to directly activate the enzyme as shown in Figure 1C. The appeal of the model shown in Figure 1C is its potentially broad generalizability to any enzyme that conforms to Koshland's induced fit model.

The utilization of substrate binding energy to drive enzyme-activating conformational changes (Figure 1C) rationalizes results obtained from studies on an eclectic set of enzymatic reactions, which include the proton transfer reaction catalyzed by TIM [17,18], the decarboxylation reaction catalyzed by OMPDC [19], the hydride transfer reaction catalyzed by GPDH [20], the complex reaction catalyzed by 1-deoxy-d-xylulose-5-phosphate reductoisomerase [21,22], and more than 50-year-old results from studies on β-phosphoglucomutase [23]. In comparison, the results of experimental work over the past 25 years have not added greatly to the extensive body of evidence for the utilization of intrinsic substrate binding energy for substrate activation cited by Jencks in his 1975 review [5].

Finally, we note that the ligand-gated conformational changes of TIM, OMPDC, and GPDH each have the effect of optimizing the stabilizing interactions between the substrate phosphoryl group and a neighboring cationic side chain: K12 for TIM; R235 for OMPDC; and R269 for GPDH. The interactions between the protein side chain cations and substrate phosphoryl group provide 7.8, 5.6, and 9.1 kcal·mol^−1^ transition state stabilization for TIM [24], OMPDC [25], and GPDH [26], respectively. In each case, this is a significant fraction of the total 12 kcal·mol^−1^ transition state stabilization achieved through interactions with the phosphoryl group of the substrate. Each of these side chain cations sits on the protein surface and forms an ion pair with the buried phosphoryl group of the substrate [26]. The placement of these side chains at the protein surface favors efficient rescue of the catalytic activity that is lost when the side chain is truncated through mutation by small-molecule analogs of the truncated side chain [14,24–26].

## Integration of experimental and computational studies

The empirical valence bond (EVB) approach [27] does an excellent job of modeling experimentally determined activation barriers for the conversion of enzyme-substrate Michaelis complexes to enzymatic transition states [28,29]. However, this approach focuses on modeling changes that occur during the actual chemical step of catalysis, and not on modeling the large-scale conformational changes upon substrate binding that can precede this step. Following from this, it is non-trivial to reliably model the free energy barrier for substrate binding that is coupled to extensive protein conformational changes, such as those observed for TIM, OMPDC, and GPDH. Despite this limitation, we have used the EVB approach with great success to rationalize the effects of ligand-gated conformational changes on the reactivity of substrates bound to TIM [30,31]. We hope to eventually generalize these calculations to model experimental results from studies on OMPDC, GPDH, and other enzymes that undergo ligand-driven conformational changes.

(1) Our proposal that the phosphite dianion-driven conformational change of TIM functions to activate this enzyme for deprotonation of enzyme-bound substrate predicts a role for the ligand-driven movement of amino acid side chains in substrate activation. We have performed EVB simulations [31], described below, to test predictions about the role of P166 [32–34], I170 [35–37], and L230 [35–38] in activation of the caged complex between enzyme and substrate for deprotonation of the carbon-acid substrate by the side chain of E165 [39,40].
(2) We proposed that the dianionic phosphoryl group of the substrate DHAP serves the exclusive role of stabilizing an enzyme-substrate cage that shows high reactivity toward deprotonation of the bound carbon acid, and predicted that the substrate dianion serves as a spectator in this cage during deprotonation of the enzyme-bound substrate [16]. This prediction requires identical activation barriers for deprotonation of the Michaelis complex to the whole substrate DHAP, and to the substrate fragments glycolaldehyde (GA) and phosphite dianion [41,42], but it was not possible to saturate TIM with the substrate fragments. This prediction was, however, confirmed by the results of EVB calculations that are described below [30].
(3) The prime imperative for the observation of effective catalysis of deprotonation of weakly acidic α-carbonyl carbon of the substrates for TIM and other enzymes is to reduce the large thermodynamic barrier for formation of the carbanion reaction intermediate [18,43,44]. The results of EVB calculations to model the effect of site-directed substitutions on the thermodynamic barrier for TIM-catalyzed deprotonation of substrates DHAP and (*R*)-glyceraldehyde 3-phosphate (GAP) provide strong evidence that the precise placement of both polar and nonpolar side chains at the caged Michaelis complex serves to minimize the thermodynamic barrier to proton transfer to the enzyme.

Finally, we have performed extensive molecular dynamics simulations, using both conventional and enhanced sampling techniques, to analyze loop motion in apo and substrate-bound TIM. The results of these studies have been combined with EVB calculations to examine the effect of displacements of loop 6 from its closed form on catalytic activity. These studies represent one step toward the full computational modeling of substrate binding to TIM [45].

## Early studies of loop dynamics and catalysis in triosephosphate isomerase

**Figure 2.**
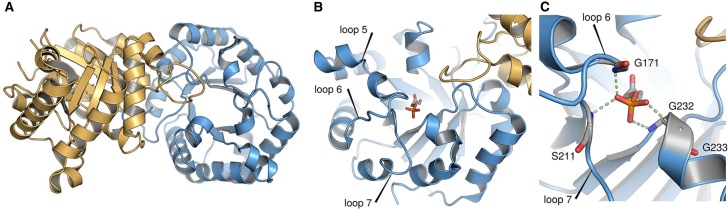
Snapshots of the structure of TIM. Overview of (**A**) the overall structure of yeast triosephosphate isomerase (*y*TIM; PDB ID: 1NEY [80,81]), showing also (**B**) key active site loops 5–7, as well as (**C**) a close up of the enzyme's active site. This figure is adapted from [45] (https://pubs.acs.org/doi/pdf/10.1021/jacs.8b09378), and is reproduced with permission from the American Chemical Society. Please note that requests for permissions regarding further reuse of this figure should be directed to the American Chemical Society.

TIM catalyzes the reversible isomerization of DHAP to GAP in the eukaryotic glycolysis pathway [18,33,46,47]. This reaction proceeds through an enzyme-bound cis-enediolate intermediate. This enzyme is an ancient TIM-barrel enzyme that likely appeared early in evolution, and TIM-barrels are both ubiquitous and highly evolvable scaffolds [48–50]. As with most TIM-barrel proteins, TIM has several key active site loops (Figure 2) that decorate the scaffold and close over the active site upon interaction with the phosphoryl group of the substrate, thus creating a hydrophobic cage in which the TIM-catalyzed reaction can take place [16,30,51]. This desolvation of the active site is critical in order to elevate the p*K*_a_ of an active site glutamic acid — E165 using *y*TIM numbering — from 3.7 in aqueous solution to >6 in the TIM active site upon substrate binding [39,40,52], allowing E165 to deprotonate a substrate with a p*K*_a_ of ∼18 [53,54].

TIM is highly proficient, with *k*_cat_/*K*_M_ values of 4.3 × 10^5 ^M^−1 ^s^−1^ and 8.4 × 10^6 ^M^−1 ^s^−1^ for the deprotonation of substrates DHAP and GAP, respectively [55]. It has been demonstrated that interactions between TIM and the phosphoryl group of the substrate GAP can account for 80% of this enzyme's rate acceleration [53,56]. Tying in with this, there have been detailed studies of the activation of TIM for hydrolysis of the substrate fragment GA by phosphite dianion (HP_i_), which have demonstrated that the binding of HP_i_ to TIM results in a ∼1000-fold increase in the second-order rate constant for the unactivated isomerization reaction of GA [(*k*_cat_/*K*_M_)_E_ to (*k*_cat_/*K*_M_)_E·HPi]_)] [8,57]. Therefore, the binding of phosphite to TIM results in a 4 kcal·mol^−1^ stabilization of the transition state for the unactivated reaction of GA [7]. It has been argued that a large fraction of the intrinsic binding energy of the phosphoryl group of the substrate (or the phosphite dianion) is used to drive the energetically demanding conformational change of the active site loops of TIM, from a catalytically unfavorable open conformation, to a catalytically competent closed conformation [7]. Notably, while the phosphate gripper loop, loop 6, undergoes a large conformational change (moving up to 7 Å as it closes over the active site), this change is coupled to more subtle but significant conformational changes in loop 7 [33]. This involves a 90° rotation of the G209–G210 peptide plane, which creates a steric clash with the P166 side chain, and which in turn triggers conformational movement along the E165–P166 plane that is critical for forcing E165 from a swung out position into the active site, in an optimal position for proton abstraction from the substrate. These motions are coupled to a substantial 180° rotation along the G210–S211 peptide bond. Therefore, while the conformational changes of loop 7 are more spatially confined than those of loop 6, they are no less critical to catalysis. Taken together, these features make TIM an ideal model system for studying the role of ligand-gated conformational changes in enzyme catalysis, while at the same time posing particular challenges for biomolecular simulations, as will be described in this contribution.

From a computational perspective, the movement of TIM loop 6 was one of the first functional enzyme motions to be investigated by means of molecule simulations [58]. In particular, Kollman [59], Karplus [60] and co-workers performed seminal molecular dynamics simulations *in vacuo*, using simple reaction co-ordinates, arguing that TIM loop closure is essentially a rigid-body type displacement. That is, they argued that the loop moves like a ‘lid’ that is attached to the protein by two hinges, the sequences of which are not conserved between different TIM-barrel proteins [61] (Figure 2). This early work has made TIM a classical system for understanding enzyme loop motion, and in fact the image of loop 6 motion as a two-state rigid-body motion is not only widespread [33,59,60,62–64], but also TIM has been used as an example of a prototype for such motion in biological systems [65]. In parallel, there have been several studies of the mechanism (including cold-adaptation) of the TIM-catalyzed reaction (Figure 1A) using a range of computational approaches [30,31,45,66–70]. Curiously, however, despite extensive computational studies of TIM, and extensive experimental evidence for the role of ligand-gated conformational changes in catalysis by TIM and other enzymes [9–12,38], computational studies have generally failed to find an activating role for such changes, in part due to the short simulation timescales and/or simplified models involved.

## Computational modeling of the role of ligand-gated conformational changes in catalysis by triosephosphate isomerase

**Figure 3.**
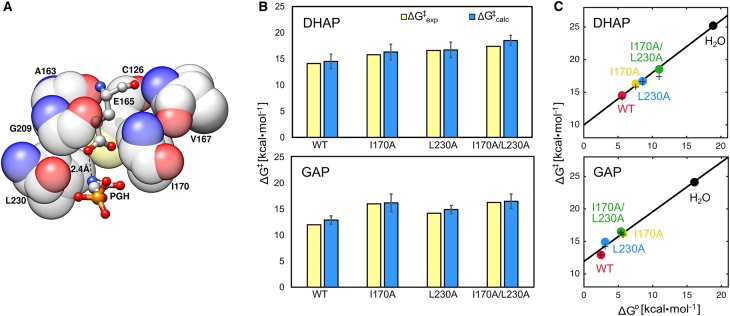
Modeling the Hydrophobic Clamp of TIM. **(A)** An illustration of the hydrophobic clamp in TIM, formed by the hydrophobic side chains of residues I170 and L230 clamping the catalytic base E165. This figure is based on the active site of wild-type *Tbb*TIM (PDB ID: 1TRD [81,82]), in complex with the intermediate analog phosphoglycolhydroxamate (PGH). (**B**) A comparison of experimental (ΔG^‡^_exp_, yellow) and calculated (ΔG^‡^_calc_, blue) activation free energies for the deprotonation of (top) DHAP and (bottom) (*R*)-glyceraldehyde 3-phosphate (GAP) by wild-type and mutant TIMs. (**C**) The correlation between the experimental (+) and calculated (●) activation free energies (ΔG^‡^), and the calculated reaction free energies (ΔG^0^) for the deprotonation of (top) DHAP and (bottom) GAP by wild-type and mutant TIMs. Here, the correlation coefficients, calculated by linear regression analysis, are 0.9987 and 0.9921 for DHAP, and 0.9898 and 0.9909 for GAP (experimental and calculated values, respectively). All energies are presented in kcal·mol^−1^. This figure is adapted from and based on data presented in ref. [31] (https://pubs.acs.org/doi/pdf/10.1021/jacs.7b05576), and is reproduced with permission from the American Chemical Society. Please note that requests for permissions regarding further reuse of this figure should be directed to the American Chemical Society.

To address the role of ligand-gated conformational changes in catalysis by TIM, as our starting point, we performed detailed EVB simulations of the mode of operation of a hydrophobic clamp (Figure 3A) in TIM [31], which acts to enhance the basicity of E165, thus facilitating efficient catalysis by this enzyme. Specifically, we considered both the TIM-catalyzed deprotonation of substrates DHAP and GAP by wild-type TIM, as well as by TIM variants with mutations of the residues forming this clamp (single and double I170A and L230A mutations, using *y*TIM numbering). Structural and biochemical characterization of these mutants in the TIM from *Trypanosoma brucei brucei* (*Tbb*TIM) [36,55] indicated diminished activity upon truncation of either side chain to alanine, while the structures of the different TIM variants in complex with the intermediate analog phosphoglycolate were nearly superimposable. The only significant difference between these structures was the presence of additional water molecules in the space made available by truncation of the hydrophobic side chains. Our EVB simulations of the different enzyme variants are able to reproduce the kinetic impact of these different mutations with excellent quantitative agreement with experiment (Figures 3B,C) [31], as well as allowing us (when combined with experimental analysis) to obtain a detailed overview of the breakdown of these changes into ground state and transition state effects. In particular, these calculations provide a linear free energy relationship (LFER), with a slope of 0.8, between the calculated activation barriers and Gibbs free energies for the TIM-catalyzed reactions studied in our work [31]. This combined experimental and computational analyses led us to conclude that the main role of these clamping side chains is to minimize the Gibbs free energy for substrate deprotonation, an effect that is largely expressed at the transition state for the TIM-catalyzed proton transfer reaction.

**Figure 4.**
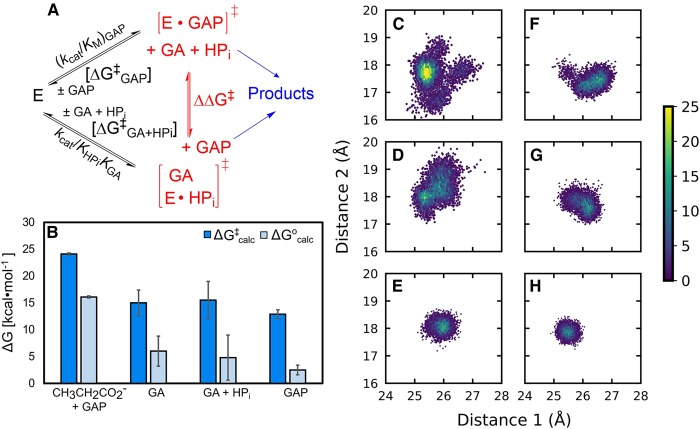
Modeling TIM-catalyzed reactions of the whole substrate and substrate fragments. (**A**) Overview of the mechanisms for the TIM-catalyzed deprotonation of the full substrate GAP and the substrate fragments glycoaldehyde and phosphite dianion (GA·HP_i_). (**B**) A comparison of calculated activation (ΔG^‡^_calc_, dark blue) and reaction (ΔG^0^_calc_, light blue) free energies for the non-enzymatic deprotonation of the full substrate GAP, as well as the corresponding TIM-catalyzed deprotonation of substrates glycoaldehyde (GA), GA·HP_i_ and GAP. All energies are shown in kcal·mol^−1^. (**C**) Population densities of the conformational space sampled by the substrate fragments (**C**,**F**) GA and (**D**,**G**) GA·HP_i_, as well as (**E**,**H**) the full substrate GAP, at (**C**–**E**) the Michaelis complexes and (**F**–**H**) transition states for the TIM-catalyzed deprotonation of these substrates, obtained from EVB simulations, performed as described in ref. [30]. The data on the *x*- and *y*-axes based on distances between the donor carbon atoms of the relevant substrate and the backbone α-amino acid carbon atoms of D111 in Chain B and I19 in Chain A. For full details of the analysis see ref. [30]. This figure is adapted from and based on data presented in ref. [30] (https://pubs.acs.org/doi/10.1021/jacs.8b00251), and is reproduced with permission from the American Chemical Society. Please note that requests for permissions regarding further reuse of this figure should be directed to the American Chemical Society.

Following from this, we extended our EVB simulations of the full substrates DHAP and GAP [31] to also modeling the energetics for the deprotonation of the substrate fragments GA and GA·HP_i_ [30] (Figure 4A). We note here that the kinetic parameters (*k*_cat_/*K*_M_, M^−1 ^s^−1^) for the TIM-catalyzed isomerization of the whole substrate GAP to form DHAP, as well as those for the corresponding phosphite dianion activation reactions of the substrate fragment GA, determined for 14 different wild-type and site-directed mutant forms of TIM, define an LFER with a slope of 1 between the activation barriers for the TIM-catalyzed reactions of the whole substrate and substrate fragments, by wild-type and mutant TIMs [41,42]. Similar relationships have been observed in other enzymes such as OMPDC [72] and GPDH [20], indicating that the primary role of the dianion activators is to stabilize the catalytically competent closed conformation of TIM (Figure 2). Our EVB simulations [30] of the deprotonation of the full substrate GAP as well as the substrate fragment GA and the fragments GA·HP_i_ yield activation free energies (ΔG^‡^_calc_) of 12.9 ± 0.8 kcal·mol^−1^ for the full substrate GAP, and 15.0 ± 2.4 and 15.5 ± 3.5 kcal·mol^−1^ for the substrate fragments GA and GA·HP_i_, respectively (Figure 4B). The increase in the activation barrier for the substrate fragments compared with the full substrate GAP is likely an entropic effect, due to the increased conformational space of the substrate fragments in the TIM active site, even in the presence of phosphite dianion (Figure 4C–H). However, the overall effect of the bound dianion on ΔG^‡^_calc_ is small at ≤2.6 kcal·mol^−1^, compared with the much larger intrinsic binding energies of 12.8 and 5.8 kcal·mol^−1^ for the phosphoryl group of the substrate and the phosphite fragment that are utilized to stabilize the transition states for the TIM-catalyzed deprotonation of GAP and GA·HP_i_, respectively [7,56]. This suggests that the dianion binding energy is fully expressed at the Michaelis complex, where it is utilized to drive an energetically unfavorable conformational change, and is the first computational evidence for the role of a ligand-gated conformational change in enzyme catalysis [30].

**Figure 5.**
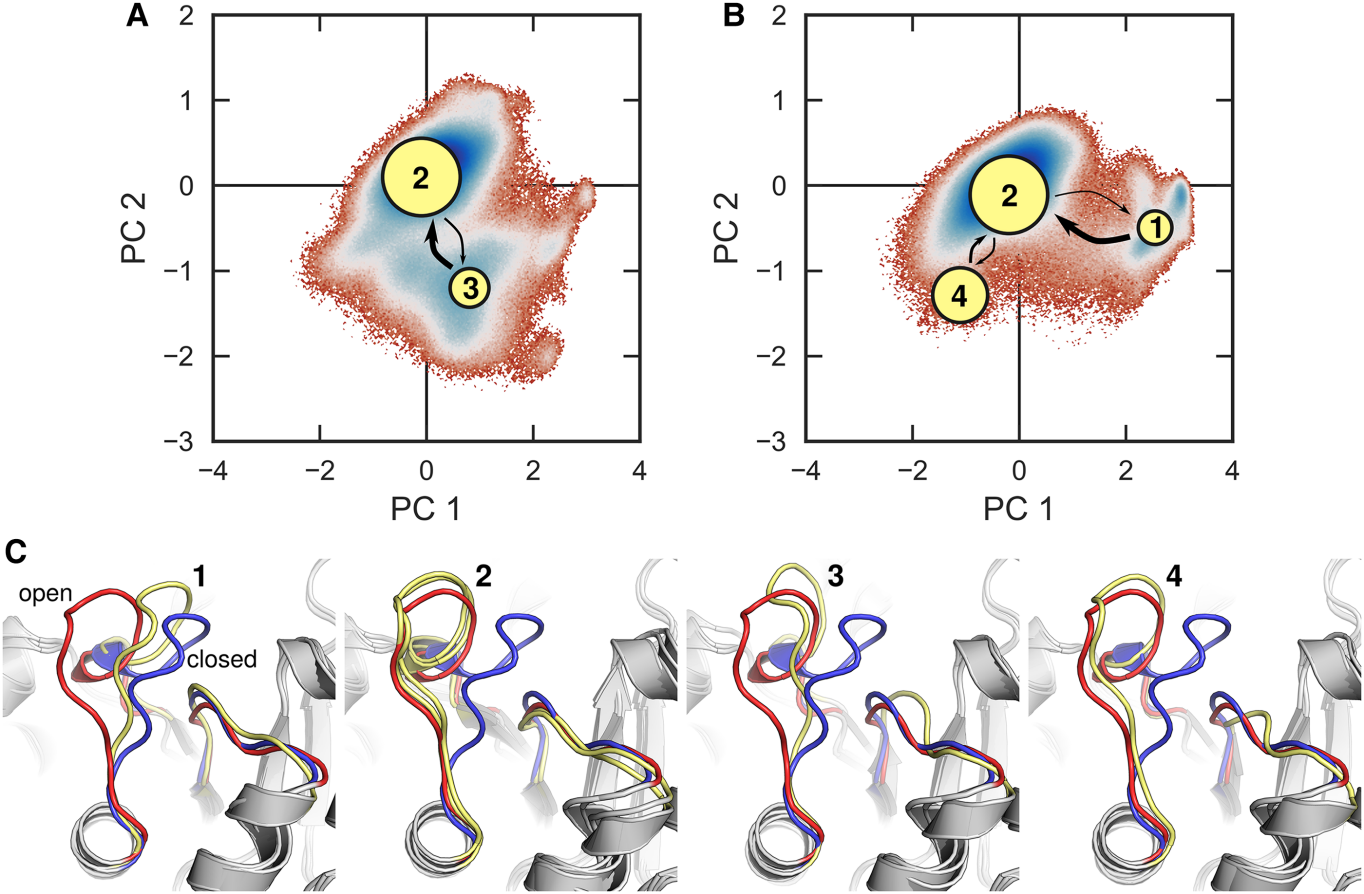
Modeling the open and closed conformations of TIM. Markov state models (MSM) of (**A**) substrate-free TIM and (**B**) TIM in complex with substrate DHAP, superimposed on free energy surfaces defined in terms of the first two principal components, PC1 and PC2, obtained from conventional molecular dynamics simulations of each system (60 independent simulations, to a total of 22 μs of simulation time). The population of each node corresponding to a metastable state is represented through the area of the node, while the thickness of the arrows connecting the node corresponds to the transition probabilities, which are shown in the Supporting Information of ref. [45]. (**C**) A comparison of the crystal structures for TIM with open (red, PDB ID: 1YPI [71,81]) and closed (blue, PDB ID: 1NEY [80,81]) conformations of loop 6, as well as representative structures from the different centroids shown in panels (**A**,**B**). The numbering corresponds to the numbering of the different centroids. It can be seen here that the substrate-bound enzyme samples a semi-closed conformation (**1**), both systems sample an open conformation that is common to the two systems (**2**), while each samples a third open conformation that is unique to each system [(**3**) in the case of the substrate-free enzyme, (**4**) in the case of the substrate-bound enzyme]. For simulation details, see ref. [45]. This figure was originally presented in ref. [45] (https://pubs.acs.org/doi/pdf/10.1021/jacs.8b09378), and is reproduced with permission from the American Chemical Society. Please note that requests for permissions regarding further reuse of this figure should be directed to the American Chemical Society.

Finally, we have also been very interested in the motion of loop 6 itself. As described above, historically this loop motion has been described as a prototypical example of a two-state rigid-body motion [33,59,60,62–64]; however, these conclusions have in large part been based on short simulation timescales and/or simplified computational models. We recently performed detailed computational studies combining both microsecond conventional molecular dynamics simulations and enhanced sampling simulations of the conformational transitions of loop 6, using five crystal structure of the dimeric TIM from *Saccharomyces cerevisiae* (*y*TIM), coupled with EVB simulations [27,29] of the associated chemical step of catalysis [45]. We found that reliably modeling loop 6 motion is highly challenging and requires advanced sampling methods in order to be able to capture the closed conformation of the loop. These calculations demonstrated that contrary to being a rigid-body motion, loop 6 is in fact highly flexible, and samples multiple conformational states in the open conformation (Figure 5). However, the closed conformation was dominated by a single conformation of loops 6 and 7, that our simulations demonstrated to move in a concerted fashion in agreement with previous structural analysis [33]. However, despite sampling multiple open conformations, our EVB simulations [45] of the TIM-catalyzed deprotonation of DHAP at different conformations of loop 6 demonstrated that even slight displacements from the fully closed crystallographically observed loop conformation can be sufficient to abolish most of the catalytic activity of TIM; therefore, while the loop is overall flexible, full loop closure is required for efficient catalysis [16,45].

Finally, the importance of loop 6 flexibility to efficient catalysis can also be seen in mutational studies of rare variations of position 96 (proline in the wild-type) of the TIM from *Plasmodium falciparum* (*Pf*TIM) [73], which is analogous to the proline at position 168 in *Tbb*TIM [34]. In both cases, it is clear that this residue plays an important role in modulating the conformational transition of loop 6, and, most critically, in positioning E165 (*y*TIM numbering) in a catalytically competent position in the TIM active site (Figure 2).

## Loop motion and dimer assembly in triosephosphate isomerase

As shown in Figure 2, in the majority of cases, TIM is structurally a homodimer, and lends its name to the archetypal TIM-barrel fold comprising of eight α-helices and eight parallel β-sheets alternating along the protein backbone [45,48,49]. However, there do exist exceptions to this: in some organisms, TIM takes on instead a tetrameric form, and it has been argued that this change in oligomerization state can be functionally important [74–76]. In particular, the tetrameric form has been argued to provide more stability to the overall scaffold, allowing the enzyme to function at extremes of temperature [74,75]. Tying in with this, recent EVB studies comparing the temperature dependence of the TIM from the psychrophilic bacterium *Vibrius marinus* as well as yeast TIM indicated a shift in the enthalpy–entropy balance between the two enzymes, which was found to be due to greater mobility of key surface loops in the cold-adapted enzyme [70]. Therefore, scaffold rigidification through changes in oligomerization state could indeed plausibly allow the enzyme to withstand higher temperatures.

Principle component analysis on a range of TIM structures has shown that the TIM structure space can be divided into two groups: open and closed TIM structures, with a greater the distribution of structures in the open set than in the closed set [76]. This is supported by all-atom simulations of TIM loop motion which indicate that while TIM loop 6 is flexible and can take on multiple open conformations, the conformational space of the closed conformation of the loop is much more restricted [45]. In addition, elastic network modeling of monomeric, dimeric and tetrameric TIMs from different organisms demonstrated that oligomerization not only stabilized the structures, but also enhanced their functional loop motions (in particular of loops 6 and 7) [76]. Following from this, elastic network models have also shown that TIM-barrel proteins with conserved structure but without functional conservation can also have greatly different intrinsic dynamics tying in with their functional differences [77]. This is not a recent evolutionary event in TIM development, as studies of ancestral TIMs obtained through ancestral inference have indicated the presence of early evolutionary coupling between oligomerization and function [78]. Finally, mutagenesis studies of TIMs from the protozoan parasite *Trichomonas vagina* (*Tv*TIM) that are capable of dissociating into stable monomers, but that dimerize once substrate binds, have shown that the dimerization is necessary for assembly of a catalytically competent active site, but that as it is being assembled, the active site itself acts to stabilize the dimer [79]. Therefore, there is clear evidence for an interplay between global conformational changes, local loop motions, and TIM function, culminating in the critical importance of ligand-gated conformational changes to drive catalysis by these enzymes.

## Concluding remarks

There is a tremendous wealth of biochemical and structural information that points to an important role for ligand-gated conformational changes in enzyme catalysis, in a wide range of enzymatic systems [13,21–23]. These studies demonstrate that ligand-gated conformational changes are one hallmark of the extraordinary efficiency of enzyme catalysis, compared with catalysis by small molecules in water. The binding energy of the phosphoryl group of the substrate that drives these conformational changes is utilized to mold enzymes into a high-energy catalytic form, and comparison of the structures of the open and closed forms of TIM has helped to identify the catalytic side chains that activate the TIM-bound substrate for a chemical reaction.

More recently, increases in computational power have allowed for both enzyme conformational changes and the fine details of the chemical mechanism to be studied in unprecedented detail. This contribution discusses examples of such computational studies, using TIM as a model system, that provide insight into the chemical step of catalysis by TIM [30,31], the role of loop conformational changes in driving the TIM-catalyzed reaction [45], and the role of oligomerization in allowing for TIMs to withstand extreme temperature conditions [76]. In particular, recent work on TIM loop motion [45] as well as the activation of the TIM-catalyzed deprotonation of the substrate fragment GA by phosphite dianion [30] show clear computational evidence for a role for the binding of anionic groups in driving a catalytically unfavorable conformational change. While our focus here has been on computational studies of a single enzyme, we have highlighted in this contribution the fact that analogs experimental data exists for a broad range of systems [9–13,15,16,20,72], and therefore we believe the conclusions from these computational studies can be extended to any enzyme that is activated by a ligand-gated conformational change. Finally, we demonstrate here how close synergy between theory and experiment is crucial for solving challenging questions with regard to our understanding of enzyme catalysis. This allows for even more challenging problems to be addressed, as described in the Outstanding Questions box.

**Perspectives**

- Enzymes are biological catalysts, which accelerate the rate of chemical reactions to levels necessary to support life. An understanding of their mechanism of action is critical to our understanding or all living systems.
- The experimental activation barrier to conversion of enzyme-substrate complexes to products have been successfully modeled using high-resolution protein crystal structures obtained by X-ray crystallographic analysis. The structures of unliganded enzymes in water often show flexible elements that undergo large conformational changes, which are driven by substrate binding to the protein. Several models have been developed to rationalize possible contributions of protein motions to enzymatic rate accelerations. A recent model emphasizes this role within the context of a general imperative for the coexistence of flexible structures for unliganded proteins and more rigid structures for their reactive Michaelis complexes.
- There is tremendous potential for experimental and theoretical studies to define mechanisms for enzyme action by characterizing the role of ligand-driven conformational change in activating enzymes for chemical catalysis.

## Abbreviations

DHAP: dihydroxyacetone phosphate
EVB: empirical valence bond
GA: glycoaldehyde
GAP: (*R*)-glyceraldehyde 3-phosphate
GPDH: glycerol 3-phosphate dehydrogenase
HP_i_: phosphite dianion
LFER: linear free energy relationship
MSM: Markov state models
OMPDC: orotidine 5′-monophosphate decarboxylase
PGH: phosphoglycolhydroxamate
TIM: triosephosphate isomerase
